# Linalool exerts antiemetic effects via dopaminergic and serotonergic pathways: Evidence from chick model and molecular docking

**DOI:** 10.1007/s00210-025-04631-9

**Published:** 2025-10-20

**Authors:** Abdullah Al Fahmi, Khadija Akter, Imam Hossen Rakib, Sakib Hossain, Ali G. Alkhathami, Md. Sakib Al Hasan, Rakib Hossan, Iffat Ara, Md. Mizanur Rahaman, Muhammad Torequl Islam

**Affiliations:** 1 Department of Pharmacy, Gopalganj Science and Technology University, Gopalganj, 8100 Bangladesh; 2 Bioinformatics and Drug Innovation Laboratory, BioLuster Research Center Ltd., Gopalganj, 8100 Bangladesh; 3 Department of Clinical Laboratory Sciences, College of Applied Medical Sciences, King Khalid University, P.O. Box 61413, 9088 Abha, Saudi Arabia; 4 Biomedical Sciences and Molecular Biology, College of Medicine and Dentistry, James Cook University, Townsville, QLD 4811 Australia; 5 Pharmacy Discipline, Khulna University, Khulna, 9208 Bangladesh

**Keywords:** Linalool, D_2_ receptor, 5HT_3_ receptor, Antiemetic activity, Molecular docking

## Abstract

Linalool (LIN), a naturally occurring monoterpene alcohol found in aromatic plants, exhibits diverse pharmacological properties, yet its antiemetic potential remains underexplored. In this study, we investigated the antiemetic efficacy of LIN using both in vivo and in silico approaches. Emesis was induced in chicks via oral administration of copper sulfate pentahydrate (50 mg/kg), and LIN was tested at doses of 25, 50, and 100 mg/kg. Its effects were compared against the standard antiemetics domperidone (DOM, 7 mg/kg) and ondansetron (OND, 5 mg/kg). Results demonstrated that LIN at 100 mg/kg significantly prolonged the emetic latency and reduced the number of retches. Notably, co-administration of LIN (50 mg/kg) with DOM significantly (*p* < 0.05) produced the most potent effect, yielding the highest latency and lowest number of retches, reflecting a synergistic interaction. Molecular docking studies revealed a strong binding affinity of LIN to the dopamine D2 receptor (− 6.4 kcal/mol) and moderate binding to the 5-HT3 receptor (− 5.3 kcal/mol), suggesting involvement of both dopaminergic and serotonergic mechanisms. These findings collectively indicate that LIN possesses significant antiemetic activity and may offer a plant-derived alternative for emesis control.

## Introduction

A complicated reaction called emesis, which typically involves increased salivation before it starts with involuntary retching, permits an animal or human to expel poisons or toxins from their system (Popa et al. 2020). It is a typically unpleasant condition that causes the stomach contents to be expelled via the mouth and is unmistakably related to gastrointestinal motor activity (Hall and Driscoll 2005). The emesis reflex, which is coordinated by the medullary vomiting center and is directly impacted by afferent innervation, the chemoreceptor trigger zone, and other parts of the central nervous system, is a reflex that occurs when a person vomits. Gastrointestinal problems are the most frequent reasons for emesis. The pathophysiology of emesis can involve practically all organs and systems, making it challenging in some instances to identify the underlying condition (Popa et al. 2020). Vomiting and nausea may happen simultaneously or separately, and this may be caused by different pathophysiologic causes. Although they are particularly successful for treating vomiting, more recent antiemetic medications like 5-Hydroxytryptamine 3 (5-HT_3_) and Neurokinin 1 (NK1) receptor antagonists are less effective for treating nausea (Heckroth et al. 2021). Emesis usually precedes nausea, which is a subjective sensation influenced by various factors. Defining nausea in animals is challenging since they cannot express it verbally, making its existence in them a debated topic (Kenward et al. 2017). Nausea and vomiting are common digestive problems that can be brought on by various emetic stimuli that reach the central and peripheral nervous systems. When hazardous chemicals, medications, germs, viruses, or fungi enter the body via the blood, skin, or respiratory systems, or through enteral (e.g., the gastrointestinal tract), nausea and vomiting are thought to be defensive mechanisms (Zhong et al. 2021). Furthermore, many adverse reactions to radiation therapy and cancer treatments might cause emesis (Wang et al. 2022).

The most commonly used antiemetic drugs for emesis are dopamine receptor antagonists (prochlorperazine, metoclopramide, and domperidone), 5-HT_3_ receptor antagonists (ondansetron, granisetron, and tropisetron), histamine receptor antagonists (cyclizine and diphenhydramine), muscarinic receptor antagonists, and neurokinin-1 receptor antagonists (Heckroth et al. 2021). In several investigations, DOM has been linked to ventricular arrhythmias, QT prolongation, and sudden cardiac death (Field et al. 2019). The dopamine antagonist metoclopramide is linked to weariness and drowsiness, and prolonged use may result in extrapyramidal symptoms such as tardive dyskinesia (Al‐Saffar et al. 2019; Radhakrishnan et al. 2020; Kalas et al. 2023). Bilious and non-bilious kinds of emesis fall into two main groups. Bile is evacuated with the stomach’s contents in bilious emesis. The bulk of the bile flows into the more distal sections of the intestine. In non-bilious vomiting, antegrade intestinal flow is intact, and some minor intestinal reflux into the stomach is frequent with any vomiting. Non-bilious emesis is typically brought on by inflammatory or infectious diseases such as acute gastroenteritis, labyrinthitis, and pancreatitis (Ahmed et al. 2013). Despite the effectiveness of current treatments, many patients continue to suffer from refractory or breakthrough emesis, especially in chemotherapy-induced and postoperative nausea and vomiting (Navari 2013). There is a pressing desire to develop a new, cost-effective antiemetic agent with stronger efficacy and fewer side effects.

Natural substances have become useful in current treatment strategies because of their few side effects and considerable medicinal benefits (Rakib et al. 2025; Al Hasan et al. 2025; Islam et al. 2025a). The possibility for creating novel antiemetic drugs is being investigated for natural materials like alkaloids, flavonoids, cannabinoids, diarylheptanoids, glycosides, polysaccharides, terpenes, and saponins (Hasan et al. 2024). Peppermint (*Mentha piperita*), used as a supplementary medication, reduces nausea, vomiting, and anorexia in breast cancer patients undergoing chemotherapy (Jafarimanesh et al. 2020). Gingerol from ginger (*Zingiber officinale*) has shown efficacy in relieving symptoms of sickness due to motion and chemotherapy-induced nausea (Crichton et al. 2019). Quercetin has promising antiemetic capabilities via regulating key receptors implicated in the vomiting response, such as the dopamine and serotonin receptors (Chowdhury et al. 2023).

LIN is a naturally produced monoterpene alcohol present in many flowers, including lavender (Aprotosoaie et al. 2014). LIN, an essential fragrance component utilized in a variety of foods, drinks, and personal hygiene products, has a pleasant flowery aroma (perfumes, body lotions, etc.). (Bauer et al. 2008). LIN is a natural compound derived from the plant Coriandrum sativum (Khalid et al. 2024). LIN exhibits diverse pharmacological activities. It possesses analgesic, antimicrobial, anti-inflammatory, anticancer, and antioxidant properties (Kamatou and Viljoen 2008; Aprotosoaie et al. 2014). In cancer research, LIN has been found to induce apoptosis and cell cycle arrest in tumor cells, suggesting its potential as an anticancer agent (Rodenak-Kladniew et al. 2018). Additionally, it plays a role in metabolic dysfunction-associated steatotic liver disease (MASLD) by improving lipid metabolism and reducing oxidative stress (Barbhuiya et al. 2025).

This study evaluates the anti-emetic effect of LIN against copper sulfate pentahydrate-induced emesis in chicks and explores its potential mechanism using a co-treatment model. Additionally, an in silico study was undertaken to examine molecular interactions that could be relevant for the observed effect.

## Materials and methods

### In vivo study

#### Chemicals and reagents

**Fig. 1:**
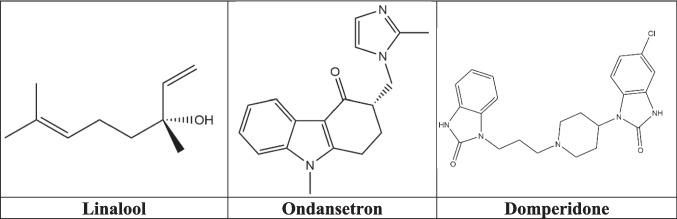
The 2D chemical structures of linalool, ondansetron, and domperidone

The compound LIN [3, 7-dimethylocta-1, 6-dien-3-ol], with a purity of 98%, and a CAS number of 78–70-6, was acquired from Sigma-Aldrich (Germany). CuSO_4_·5H_2_O and Tween 80 were obtained from Merck (Mumbai, India). The reference/standard drugs OND and DOM were obtained from Incepta Pharmaceuticals Ltd. (Dhaka, Bangladesh) and Beximco Pharmaceuticals Ltd. (Dhaka, Bangladesh), respectively. The 2D chemical structures of LIN, OND, and DOM are shown in Fig. 1.

#### Experimental animals

Country of origin (*Gallus gallus domesticus*) chicks (40–42 g) of 2-days-old of either sex were purchased from Rupa Poultry Farm and were maintained at the Pharmacology Lab of Gopalganj Science and Technology University (GSTU), Gopalganj, for the present study. The animals were allowed free access to standard food and water ad libitum. They were kept under controlled lighting (12 h dark/light cycle) at 27 ± 1 °C until the test commenced. The present experiment was conducted from 08:00 am to 3:00 pm, and the animals were monitored for 17 h to check their possible mortality after the study. Experimental design and procedures were approved by the Department of Pharmacy at the GSTU (#gstu/phr1-90/19).

#### In vivo protocols

**Fig. 2:**
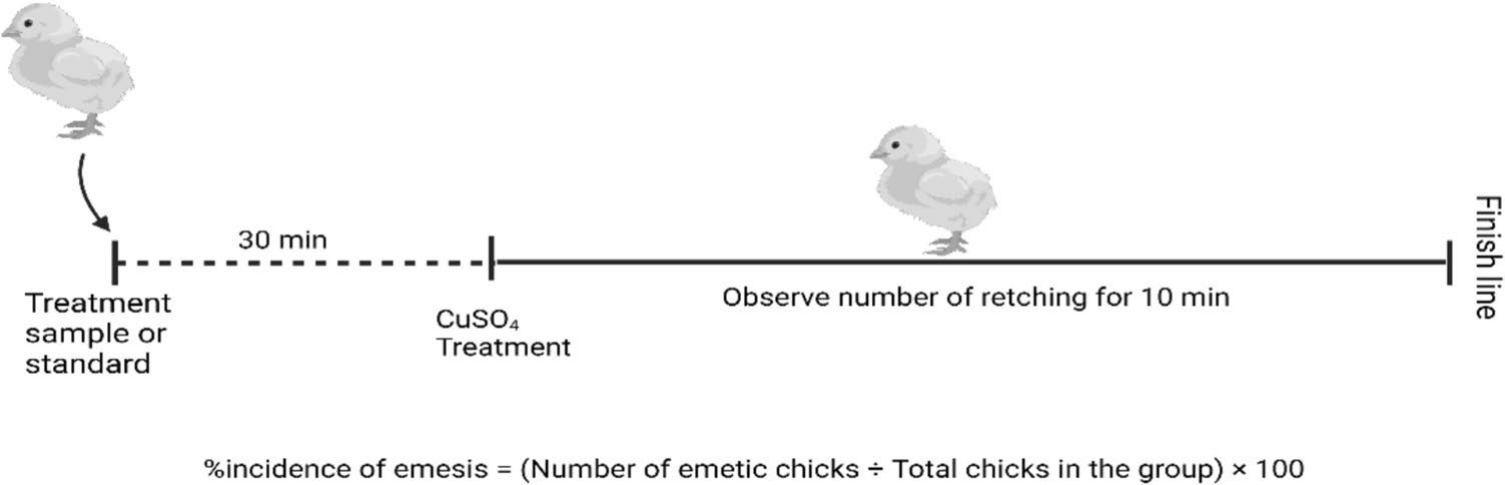
Work outline of anti-emetic test in chicks

This study was conducted according to the method described by Akita et al. (1998) with slight modifications illustrated in Fig. 2. Briefly, after one-day acclimation, chicks of either sex are divided into groups shown in Table 1. The chosen sample size was determined based on prior literature and ethical considerations to minimize animal use in compliance with the 3Rs principle (Replacement, Reduction, and Refinement) (Afroz et al. 2024). The animals were treated accordingly thirty minutes before CuSO_4_ (50 mg/kg, p.o.) administration, and each chick was kept in a large plastic jar or glass beaker at ambient temperature for 10 min. Then, the number of first retches and total retches within 10 min was counted. The latency and total retches were counted. Then, the percentage incidence of emesis and reduction of emesis were determined as follows:

$$\text{Reduction in retches }\left(\mathrm{\%}\right)=100\times \frac{{\mathrm{R}}_{\mathrm{control}}-{\mathrm{R}}_{\mathrm{treatment}}}{{\mathrm{R}}_{\mathrm{control}}}$$

where, R_control_ = mean number of retches in the control group, and.

**Table 1:** Name of treatments, their dose, and target receptor

Treatment groups	Composition	Dose	Target receptor
Control	Control (0.5% Tween 80 dissolved in normal saline)	10 ml/kg	-
LIN-25	Linalool	25 mg/kg	Under investigation
LIN-50		50 mg/kg	
LIN-100		100 mg/kg	
OND-5	Ondansetron	5 mg/kg	5-HT_3_
DOM-7	Domperidone	7 mg/kg	D_2_
LIN-50 + OND-5	Linalool + Ondansetron	50 + 5 mg/kg	Under investigation
LIM-50 + DOM-7	Linalool + Domperidone	50 + 7 mg/kg	Under investigation

All treatments are given at 10 ml/kg via oral gavage (p.o.); *LIN-25* Linalool 25 mg/kg; *OND-5* Ondansetron 5 mg/kg; *DOM-7* Domperidone 7 mg/kg

R_treatment_ = mean number of retches in the treatment group.

$$\text{Incidence of emesis }\left(\mathrm{\%}\right)=100\times \frac{{\mathrm{N}}_{\mathrm{emetic}}}{{\mathrm{N}}_{\mathrm{total}}}$$

where, N_emetic_ = number of animals showing at least one retch, and.

N_total_ = total number of animals in the group.

#### Statistical analysis

The results are presented as mean ± SD (standard deviation) or percentage. The data are analyzed by means of analysis of variance (ANOVA) followed by *t*-Student–Newman–Keuls’s as a post-hoc test using the statistical software GraphPad Prism (version 6.5, origin), and experimental groups are compared with the control group. The levels of statistical significance ranged with *p* < 0.05 at 95% confidence intervals.

### In silico investigation

#### Receptor selection and preparation

Based on existing literature, we identified and selected two receptors to conduct molecular docking and visualize ligand-receptor interactions. From the Protein Data Bank (PDB) (https://www.rcsb.org/), we retrieved the three-dimensional (3D) structures of the targeted receptors, namely D_2_ (PDB ID: 6CM4) and 5HT_3_ (PDB ID: 6Y5B) (Chowdhury et al. 2023). After obtaining the receptors, the PyMol software (v2.4.1) was employed to eliminate unnecessary molecules, including lipids, heteroatoms, and water molecules, from the protein structure. This step was taken to refine the receptors and avoid potential interference during docking. Subsequently, the SwissPDB Viewer software, along with the GROMOS96 force field, was used to optimize the receptor’s geometry and energy. The final PDB file was saved for further use in the molecular docking process (Jahan et al. 2025).

#### Ligand collection and preparation

The PubChem chemical database (https://pubchem.ncbi.nlm.nih.gov/) provided structure-data file (SDF) format for the 3D conformers of DOM (Compound CID: 3151), OND (Compound CID: 4595), and LIN (Compound CID: 6549). Chem3D, a popular tool for molecular docking investigations, was used to improve the 3D conformers of chemical agents to minimize internal energy (Islam et al. 2024).

#### Molecular docking protocol

Molecular docking is a computational method widely used in medicinal chemistry for drug design. To evaluate the binding potential of drug molecules to the active sites of receptors, docking was performed with PyRx (v0.8) using AutoDock Vina. For the docking process, the grid box dimensions were set to their maximum values along the x, y, and z axes, and the calculations were performed over 200 steps. The docking results were saved in comma-separated values (CSV) file format, while the ligand–protein complex was generated in PDB format and converted to PDBQT format for further analysis. The interactions between the ligand and receptor, along with their active sites, were analyzed using Discovery Studio Visualizer (v21.1.020298) and PyMol (v2.4.1) software. Additionally, details such as amino acid (AA) residues, bond types, the number of hydrogen bonds (HB), the length of HBs, and other bond types for each ligand-receptor interaction were recorded (Stanzione et al. 2021; Islam et al. 2025b).

#### Toxicity prediction

We investigated the toxicity profiles of chemical compounds and traditional pharmaceuticals using online web servers to evaluate the safety of drugs or substances. This involved analyzing various toxicity endpoints, including carcinogenicity, nephrotoxicity, hepatotoxicity, cardiotoxicity, cytotoxicity, neurotoxicity, LD_50_ values, and toxicity classifications. To predict the toxicity of each molecule, we retrieved its canonical SMILES from the PubChem database (https://pubchem.ncbi.nlm.nih.gov/, accessed on 19 March 2025) and input it into the parameter analysis search box of the ProTox-3.0 tool (Bappi et al. 2024).

## Results

### In vivo findings

The control group exhibited a latency of 0.78 ± 0.11 min. All treatment groups significantly (*p* < 0.05) prolonged latency compared to the control. LIN-25 (3.10 ± 0.93 min), LIN-50 (6.24 ± 2.23 min), and LIN-100 (7.13 ± 3.61 min) demonstrated a dose-dependent increase in latency, with LIN-100 showing the highest prolongation, significantly (*p* < 0.05) greater than LIN-25 and comparable to LIN-50, OND-5 (5.50 ± 2.77 min), and DOM-7 (6.35 ± 2.92 min).

On the other hand, in the control group, the number of retches was 56.60 ± 2.51. All treatments significantly (*p* < 0.05) reduced retches compared to control. LIN-25 (2.4 ± 0.24) and LIN-50 (2.2 ± 0.37) exhibited profound reductions, with LIN-50 slightly more effective. LIN-100 (5.6 ± 3.1) was also effective but less so than LIN-25 and LIN-50. OND-5 (4.0 ± 1.70) and DOM-7 (10.6 ± 5.30) significantly (*p* < 0.05) reduced retches but were less effective than LIN-25 and LIN-50.

The LIN-50 + OND-5 combination yielded 6.50 ± 1.26 min latency and 22.4 ± 1.63 retches. Compared to control, this combination prolonged latency and reduced retches; however, the number of retches remained substantially higher than with LIN alone or OND-5 alone, and far higher than the LIN-50 + DOM-7 combination (1.4 ± 0.97). Therefore, no synergy was observed for LIN + OND; the effect appeared sub-additive relative to the single agents. Overall, all active treatments increased latency and reduced retches compared to control, with the strongest effects observed for LIN-25, LIN-50, and LIN-50 + DOM-7, followed by OND-5, LIN-100, and DOM-7, whereas LIN-50 + OND-5 showed the weakest reduction among active groups. The latency period and number of retches of all the treatment groups are exhibited in Table 2.

**Table 2:** Latent period and number of retches observed in test and/or control groups

Treatment groups	Latency (Min)	Number of Retches
Control	0.78 ± 0.11	56.60 ± 2.51
LIN-25	3.10 ± 0.93^*^	2.4 ± 0.24^*abcd^
LIN-50	6.24 ± 2.23^*ad^	2.2 ± 0.37^*abcde^
LIN-100	7.13 ± 3.61^*abdef^	5.6 ± 3.1^*ab^
OND-5	5.50 ± 2.77^*a^	4.0 ± 1.70^*abc^
DOM-7	6.35 ± 2.92^*abd^	10.6 ± 5.30^*a^
LIN-50 + OND-5	6.50 ± 1.26^*abde^	22.4 ± 1.63^*^
LIN-50 + DOM-7	7.82 ± 2.01^*abcdef^	1.4 ± 0.97^*abcdef^

Values are Mean ± SD (standard deviation) (n = 5); One-way ANOVA followed by *t*-Student–Newman–Keuls’s as post-hoc test; ^*^p < 0.05 compared to the control group; ^a^*p* < 0.05 compared to the LIN-25; ^b^*p* < 0.05 compared to the LIN-50; ^c^*p* < 0.05 compared to the LIN-100;^d^*p* < 0.05 compared to the OND-5; ^e^*p* < 0.05 compared to the DOM-7;^f^*p* < 0.05 compared to the LIN-50 + OND-5; ^g^*p* < 0.05 compared to the LIN-50 + DOM-7; LIN-25: Linalool 25 mg/kg; LIN-50: Linalool 50 mg/kg; LIN-100: Linalool 100 mg/kg; OND-5: Ondansetron 5 mg/kg; DOM-7: Domperidone 7 mg/kg

Percentage reduction analysis showed that LIN-25, LIN-50, and LIN-100 reduced emesis by 95.86%, 96.20%, and 90.34%, respectively. OND-5 and DOM-7 reduced emesis by 93.10% and 81.72%, while the combinations LIN-50 + DOM-7 and LIN-50 + OND-5 achieved reductions of 97.58% and 94.13%, respectively. Regarding incidence, all chicks in the control, LIN-25, and LIN-50 groups exhibited emesis (100%). Incidence was reduced to 80% in the OND-5, DOM-7, and LIN-50 + OND-5 groups, to 60% in LIN-100, and to 40% in LIN-50 + DOM-7. The percentage incidence and percentage reduction of emesis of all the treated groups are shown in Table 3.

**Table 3:** Percentage incidence and percentage reduction of emesis in test and/or control groups

Treatment groups	Reduction of emesis (%)	Emesis incidence (%)
Control	0.00	100
LIN-25	95.86	100
LIN-50	96.20	100
LIN-100	90.34	60
OND-5	93.10	80
DOM-7	81.72	80
LIN-50 + OND-5	94.13	80
LIN-50 + DOM-7	97.58	40

Values are percentage; *LIN-25* Linalool 25 mg/kg; *LIN-50* Linalool 50 mg/kg; *LIN-100* Linalool 100 mg/kg; *OND-5* Ondansetron 5 mg/kg; *DOM-7* Domperidone 7 mg/kg

### In silico investigation

#### Molecular docking and visualization of non-bond interactions between protein–ligand complexes

For the D_2_ receptor, LIN exhibited a BA of –6.4 kcal/mol, while the standard DOM compound showed a BA of –9.8 kcal/mol. LIN did not form any HB. On the other hand, DOM formed one HB with SER A: 409 AA residue. LIN’s binding interaction with the D2 receptor was facilitated by non-HBs interactions such as TRP A: 386, CYS A: 118, and so on. On the other hand, DOM’s binding interaction with non-HB interactions with ASP A: 114, PHE A: 389, ILE A: 184, and so on.

For the 5HT_3_ receptor, LIN exhibited a BA of –5.3 kcal/mol, while the standard OND compound showed a BA of − 8.5 kcal/mol. LIN’s binding interaction with the 5HT_3_ receptor was facilitated by non-HBs interactions with specific AA residues, for example, VAL A: 237, VAL E: 295, LEU A: 234, etc. On the other hand, OND’s binding interaction with the 5HT_3_ receptor was facilitated by non-HBs interactions with ASP A: 114, PHE A: 389, ILE A: 184, and so on.

**Fig. 3:**
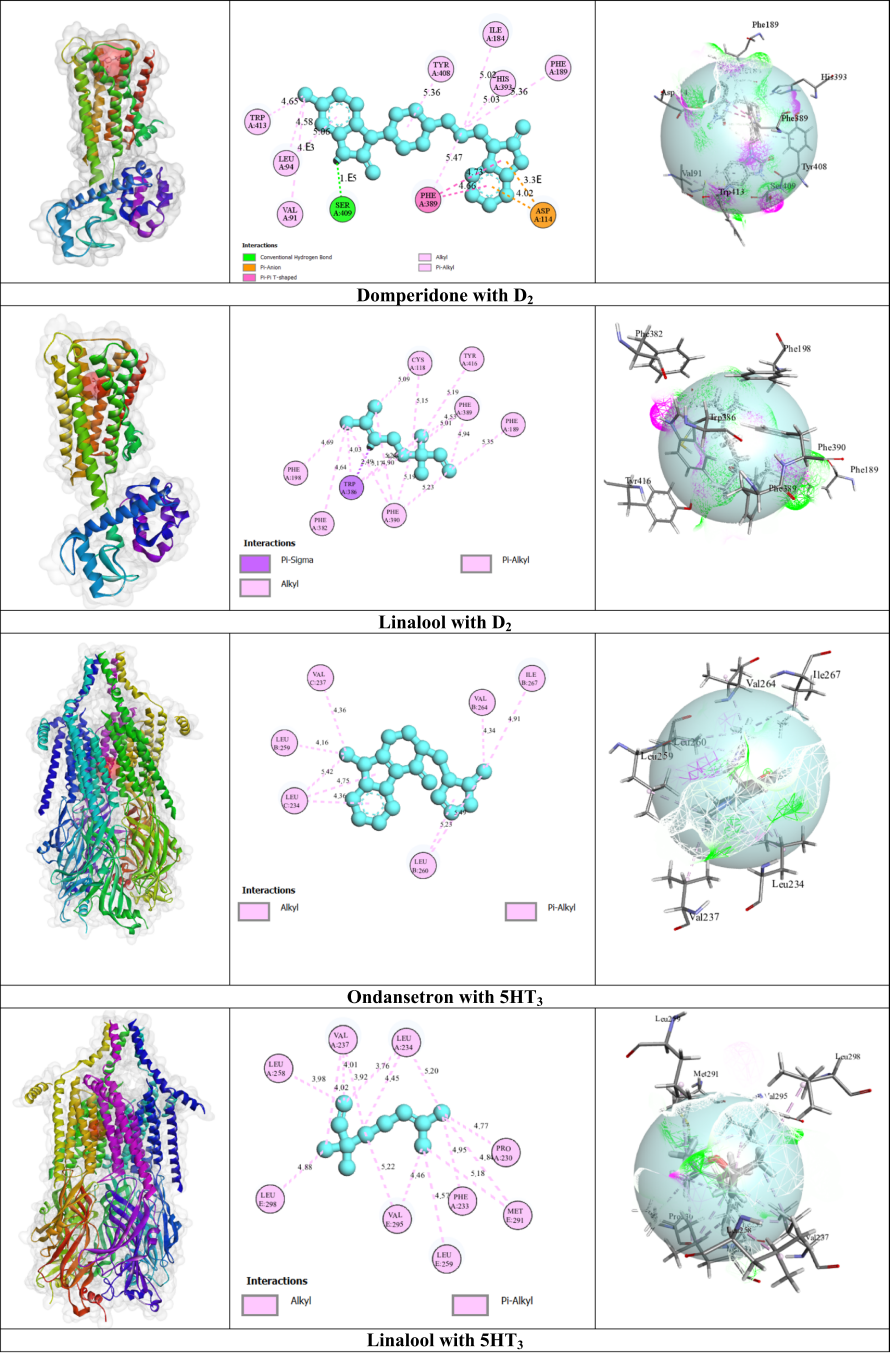
The best possible interaction for molecular docking of the D_2_ receptor with domperidone and linalool as well as 5HT_3_ receptor with ondansetron and linalool compounds [D_2_: Dopamine Receptor D_2_; 5HT3: 5-hydroxytryptamine type 3]

The overall molecular docking result is shown in Table 4. The 3D and 2D structures of the non-bond interactions between LIN and DOM with the D_2_ receptor and 5HT_3_ receptor are shown in Fig. 3.

**Table 4:** The best results of a molecular docking study of domperidone, linalool, and ondansetron with D_2_ and 5HT_3_ receptors

Ligands	Receptors (PDB ID)	BA	No of HB	Amino acid (AA) residues	
				HBs	Others
DOM	D_2_ (6CM4)	–9.8 kcal/mol	1	SER A: 409	ASP A: 114, PHE A: 389, ILE A: 184, VAL A: 91, LEU A: 94, PHE A: 189, HIS A: 393, TYR A: 408, TRP A: 413
LIN		–6.4 kcal/mol	-	-	TRP A: 386, CYS A: 118, PHE A: 198, PHE A: 382, TRP A: 386, PHE A: 390, TYR A: 416
OND	5HT_3_ (6Y5B)	–8.5 kcal/mol	-	-	LEU C: 234, LEU B: 260, VAL B: 264, ILE B: 267, LEU B: 259, VAL C: 237
LIN		–5.3 kcal/mol	-	-	VAL A: 237, VAL E: 295, LEU A: 234, LEU A: 258, PRO A: 230, MET E: 291, LEU E: 259, LEU E: 298, PHE A: 233

*BA* Binding affinity; *DOM* Domperidone; *HB* Hydrogen bond; *LIN* Linalool; *OND* Ondansetron; *D2* Dopamine Receptor D_2_; *5HT*_*3*_ 5-hydroxytryptamine type 3

#### In silico toxicity predictions

In our in silico toxicity analysis, LIN had an LD_50_ of 1190 mg/kg body weight, placing it in toxicity class 4. There were no carcinogenic, mutagenic, or cytotoxic effects associated with LIN. Nonetheless, LIN exhibited hepatotoxicity and immunotoxicity. Standard chemicals, such as DOM and OND, have been shown to be mutagenic and immunotoxic, respectively. The toxicity prediction of three linalool, domperidone, and ondansetron is shown in Table 5.

**Table 5:** The toxicity prediction of three compounds, namely linalool, domperidone, and ondansetron using the ProTox-3.0 model

Properties	Parameters	Linalool	Domperidone	Ondansetron
Toxicity	LD_50_	1190 mg/kg	715 mg/kg	95 mg/kg
	Toxicity class	4	4	3
	Hepatotoxicity	Active	Inactive	Inactive
	Carcinogenicity	Inactive	Inactive	Inactive
	Immunotoxicity	Active	Active	Inactive
	Mutagenicity	Inactive	Inactive	Active
	Cytotoxicity	Inactive	Inactive	Inactive

*LD*_*50*_ Lethal dose 50

## Discussion

Emesis, commonly known as vomiting, is a complex physiological process that involves the coordinated activation of multiple brain regions and the autonomic nervous system (Zhong et al. 2021). It is a protective reflex that helps to expel harmful substances from the body (Saito et al. 2003). Several neurotransmitter systems and receptors are involved in the emesis mechanism. The 5-HT_3_ receptor, which is a subtype of the serotonin receptor, plays a crucial role in emesis. It is located in the area postrema, which is an area in the brain that lacks the blood–brain barrier and is sensitive to circulating toxins. Activation of 5-HT_3_ receptors by serotonin or other agonists triggers emesis (Raybould et al. 2020; Zhong et al. 2021). The dopamine D_2_ receptor is also involved in emesis. It is present in the chemoreceptor trigger zone (CTZ), which is an area in the brain that detects toxins in the blood and sends signals to the vomiting center to initiate emesis. Antagonists of the D_2_ receptor, such as metoclopramide, are commonly used to prevent emesis (Belkacemi and Darmani 2020; Xu et al. 2024; Tonini et al. 2004).

The emesis model evaluates vomiting responses in animals by administering gastric irritants to induce emesis. Various species serve as experimental models for testing antiemetic drugs, including rats, ferrets, pigs, chicks, dogs, frogs, minks, pigeons, monkeys, and cats (Sharma et al. 2025). When copper sulfate is delivered orally to young chicks (*Gallus gallus domesticus*), it causes emesis. CuSO_4_.5H_2_O acts as a gastric irritant, stimulating the mucosal lining of the stomach and causing the release of serotonin (5-HT) from enterochromaffin cells. This can cause vomiting (Raybould et al. 2020). This serotonin binds to 5-HT_3_ receptors, which activate vagal afferent neurons that convey signals to the vomiting center in the brainstem, and this pathway results in the generation of emesis as a protective mechanism to evacuate the ingested harmful chemical (Rojas and Slusher 2012).

Our chosen standard drug, a D_2_ receptor antagonist such as DOM, works effectively to block nausea trigger pathways, relieving signs and symptoms through decreasing the activity of them throughout the CTZ of the nervous system (Nasrul et al. 2024). In our present study, the vehicle group displayed the highest frequency of retches, whereas the group treated with the standard drug DOM showed fewer retches, demonstrating the significant anti-emetic effects of the standard treatment. Additionally, 5HT_3_ receptors are key mediators in triggering vomiting by transmitting signals from the gastrointestinal tract. They also play a vital role in modulating intestinal motility and peristalsis through the intestinal nervous system (Guzel and Mirowska-Guzel 2022). In our study, our findings demonstrated that LIN possesses noteworthy antiemetic properties, although the magnitude of its effects varied depending on dose and co-administration. LIN at 100 mg/kg alone exhibited a pronounced antiemetic effect, producing results comparable to those observed with OND-5 and DOM-7. This indicates that LIN at higher doses has therapeutic potential as an antiemetic agent. However, the performance of LIN in combination therapy showed dose-dependent variability.

The combination of LIN-50 with DOM-7 produced the most effective antiemetic response, with the lowest number of retches recorded among all groups. This suggests a potential synergistic effect between LIN and DOM, highlighting the value of combining phytochemicals with established antiemetic drugs to achieve greater efficacy at lower doses. In contrast, the LIN-50 + OND-5 group did not demonstrate enhanced efficacy. Instead, it showed a substantially higher number of retches compared to OND-5 alone. This outcome clearly indicates that LIN may not interact positively with OND, and the combination did not provide additional benefit. Such findings underscore the importance of evaluating specific drug-phytochemical interactions, as not all combinations result in synergism.

**Fig. 4:**
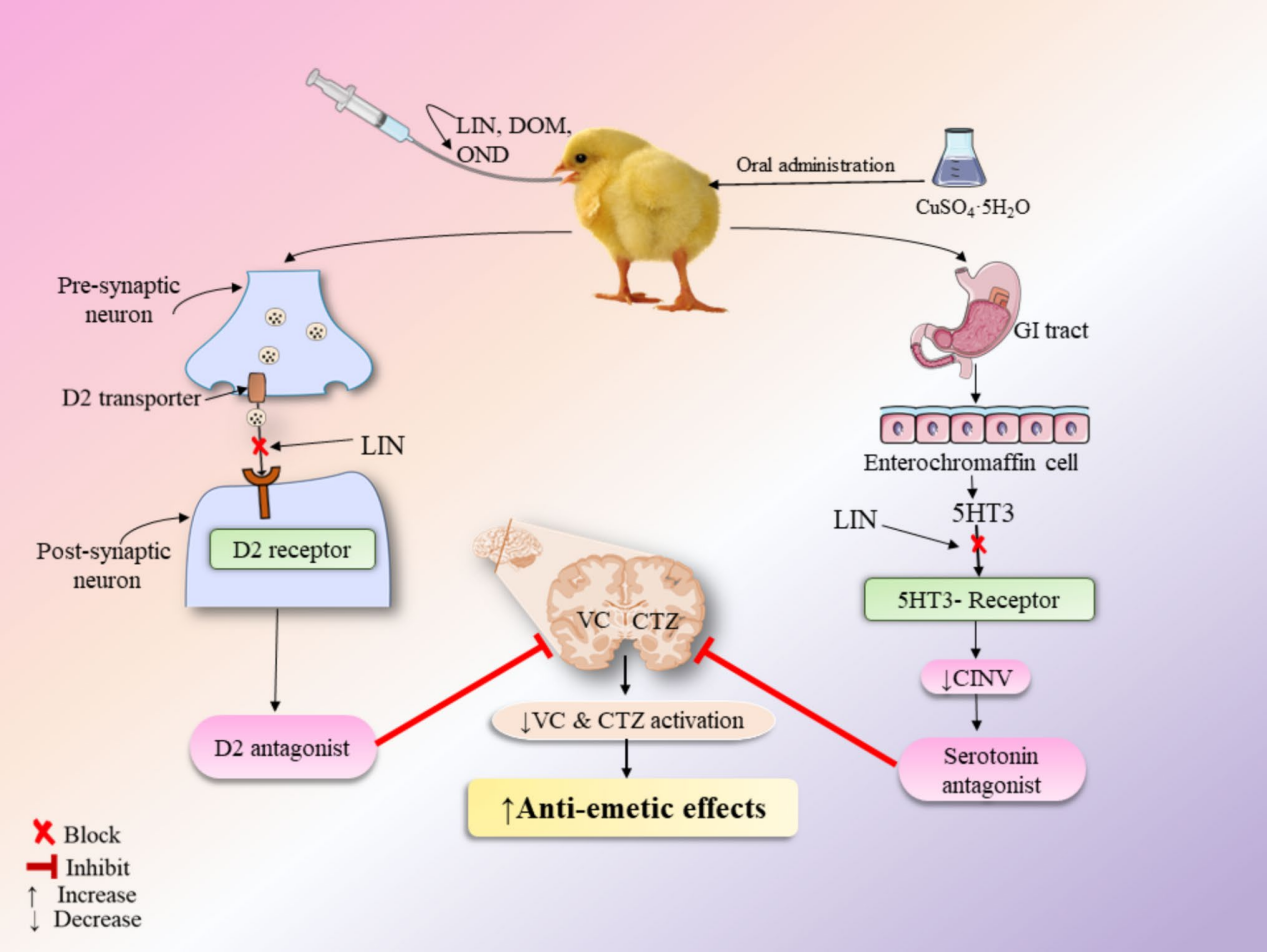
The possible antiemetic mechanism of linalool. [This figure depicts after oral administration, CuSO₄0.5H₂O triggers emesis by stimulating the gastrointestinal (GI) tract, leading to serotonin release from enterochromaffin cells and subsequent activation of 5HT3 receptors. Linalool (LIN) is proposed to act, at least in part, by modulating 5HT3 and D₂ receptor activity, thereby reducing activation of the vomiting center (VC) and chemoreceptor trigger zone (CTZ). While this figure provides a conceptual model, it should be interpreted cautiously, as not all experimental groups (e.g., LIN + OND) showed consistent efficacy, and further mechanistic studies are needed to confirm these interactions.]

Taken together, our results suggest that LIN has promising antiemetic potential, particularly at higher doses and in combination with DOM. The lack of efficacy in the LIN + OND combination highlights the complexity of pharmacodynamic interactions and emphasizes the need for further mechanistic studies to elucidate the pathways through which LIN exerts its effects. Future investigations should also assess the safety, pharmacokinetics, and receptor-level interactions of LIN, both alone and in drug combinations, to better define its therapeutic relevance in managing emesis. However, the possible antiemetic action of linalool is shown in Fig. 4.

Molecular docking is a computational approach used to predict the preferred orientation and BA of a ligand when it interacts with a target protein (Stanzione et al. 2021). It plays a crucial part in drug discovery by helping to identify and optimize potential drug candidates through predicting how small molecules bind to target proteins (Torres et al. 2019). The in silico results revealed important insights into the BA and interactions of LIN, DOM, and OND towards the D_2_ and 5HT_3_ receptors. For the D_2_ receptor, LIN exhibited moderate BA (–6.4 kcal/mol), while the standard drug DOM showed a stronger BA of –8.4 kcal/mol. On the other hand, LIN showed a moderate BA of –5.3 kcal/mol towards the 5HT_3_ receptor, while the standard DOM exhibited a stronger BA of –9.8 kcal/mol. The outcome shows that LIN exhibited a moderate BA that is closer to that of OND and DOM, which means that they can have the same effect as OND and DOM. In addition, the same amino acid residues indicate crucial binding sites, validating target engagement and supporting the test compound’s efficacy (Al-Amin et al. 2022). The common amino acid residues (PHE A: 189) in DOM and LIN suggest similar binding interactions with the 6CM4 receptor, probably leading to comparable physiological effects.

Toxicity testing increases the success rates of medication development, lowers adverse effects, and conforms to ethical research norms (Pognan et al. 2023). Our in silico toxicity analysis exhibited that LIN showed a higher LD_50_ value than standard drugs. Additionally, the tested sample’s LIN exhibited dose-dependent antiemetic efficacy in chicks. Animals showed notable antiemetic effects from LIN as compared with the standard and vehicle groups*.* Toxicity investigations indicated that LIN exhibits hepatotoxic and immunotoxic properties. Although both LIN and DOM exert antiemetic effects partly through dopaminergic modulation, their in silico toxicity predictions differ due to variations in their chemical structures, metabolic pathways, and interaction profiles with off-target proteins. DOM, a synthetic dopamine D_2_ receptor antagonist, has been strongly associated with cardiotoxicity, including QT prolongation and ventricular arrhythmias, whereas LIN, a natural monoterpene, displayed a higher LD_50_ value and lacked mutagenic or carcinogenic liabilities in our prediction model. These structural and pharmacokinetic differences likely explain the distinct toxicity outcomes despite overlapping pharmacological targets. However, an additional acute toxicity test is needed to justify and establish its toxicity profile.

Although LIN required a relatively higher dose (100 mg/kg) to exhibit its most pronounced antiemetic effect compared to conventional agents such as OND or DOM, its pharmacological profile supports its candidacy as a promising antiemetic. LIN displayed a wide safety margin, with a higher LD_50_ than both reference drugs and no mutagenic or carcinogenic liabilities, while its synergistic effect with DOM suggests that lower effective doses may be achievable in combination therapy. However, the lack of benefit observed in the LIN + OND combination, where retches were markedly higher than OND alone, highlights the complexity of drug-phytochemical interactions and indicates that LIN may not be compatible with all standard therapies.

This study also has several other limitations. The relatively small sample size (n = 5 per group) may reduce statistical power and limit generalizability; the influence of animal stomach physiology, geometry, and prior food intake could have affected emesis outcomes; and the crude form of LIN required higher doses to achieve efficacy. To address these concerns, future investigations should employ larger cohorts, evaluate receptor-specific mechanisms, and systematically study potential antagonistic or sub-additive interactions with standard agents like OND. Moreover, advanced formulation strategies such as encapsulation, controlled release systems, or timing modifications should be explored to improve absorption and minimize gastrointestinal interactions, while in vitro digestion models and comprehensive toxicological assessments will be crucial for enhancing bioavailability, safety, and translational relevance.

## Conclusion

This study demonstrates that LIN possesses notable antiemetic activity in a CuSO_4_·5H_2_O-induced chick emesis model, with efficacy comparable to standard therapeutics and synergistic effects when combined with domperidone. Molecular docking results suggest a preferential interaction of LIN with the dopamine D_2_ receptor, supporting a dopaminergic mechanism of action. However, in silico toxicity predictions indicate potential hepatotoxic and immunotoxic liabilities, warranting cautious interpretation. Further in vivo toxicological assessments and mechanistic studies are essential to validate these findings. Future research should focus on delineating LIN’s receptor-specific pathways, optimizing dosage strategies, and evaluating its translational potential for clinical use, particularly in chemotherapy or radiation-induced nausea and vomiting.

## Data Availability

Data will be provided upon request.
